# Effect of colchicine on the outcomes of patients with COVID-19: a systematic review and meta-analysis of randomised controlled trials

**DOI:** 10.1080/07853890.2022.2096919

**Published:** 2022-07-14

**Authors:** Shao-Huan Lan, Chi-Kuei Hsu, Chih-Cheng Lai, Shen-Peng Chang, Li-Chin Lu, Shun-Hsing Hung, Wei-Ting Lin

**Affiliations:** a School of Pharmaceutical Sciences and Medical Technology, Putian University, Putian, China; b Department of Internal Medicine, E-Da Hospital, I-Shou University, Kaohsiung, Taiwan; c Division of Hospital Medicine, Department of Internal Medicine, Chi Mei Medical Center, Tainan, Taiwan; d Yijia Pharmacy, Tainan, Taiwan; e School of Management, Putian University, Putian, China; f Division of Urology, Department of Surgery, Chi-Mei Hospital, Chia Li, Tainan, Taiwan; g Department of Orthopedic, Chi Mei Medical Center, Tainan, Taiwan

**Keywords:** Colchicine, COVID-19, mechanical ventilation, mortality, non-invasive ventilation, SARS-CoV-2

## Abstract

**Aim:**

This meta-analysis aimed to assess the usefulness of colchicine in patients with COVID-19.

**Methods:**

PubMed, Web of Science, Ovid MEDLINE, the Cochrane Library, Embase, and Clinicaltrials.gov were searched for relevant randomised controlled trials (RCTs) published between database inception and November 12, 2021. Only RCTs that compared the clinical efficacy and safety of colchicine with other alternative treatments or placebos in patients with COVID-19 were included.

**Results:**

Overall, 7 RCTs involving 16,024 patients were included; 7,794 patients were in the study group receiving colchicine and 8,230 were in the control group receiving placebo or standard treatment. The study and control groups had similar risk of mortality (odds ratio [OR], 1.00; 95% CI, 0.91–1.09; *I*^2^ = 0%). No significant difference was observed between the study and control groups in terms of the need for non-invasive ventilation (OR, 0.92; 95% CI, 0.83–1.03; *I*^2^ = 0%), the need for mechanical ventilation (OR, 0.64; 95% CI, 0.32–1.32; *I*^2^ = 58%), and length of hospital stay (mean difference, −0.42 days; 95% CI, −1.95 to 1.11; *I*^2^ = 62%). In addition, colchicine was associated with significantly higher risks of gastrointestinal adverse events (OR, 1.81; 95% CI, 1.56–2.11; *I*^2^ = 0%) and diarrhoea (OR, 2.12; 95% CI, 1.75–2.56; *I*^2^ = 9%).

**Conclusions:**

Colchicine does not improve clinical outcomes in patients with COVID-19, so it did not support the additional use of colchicine in the treatment of patients with COVID-19.

**Key message**
- Colchicine could not reduce the mortality of patients with COVID-19.
- No significant difference was observed between the colchicine and comparators in terms of the need for non-invasive ventilation, need for mechanical ventilation, and length of hospital stay.
- Colchicine was associated with a higher risk of gastrointestinal adverse events.

## 1. Introduction

As of May 25, 2022, more than 524 million confirmed cases of coronavirus disease 2019 (COVID-19) were reported, including more than 6 million deaths [1]. Most patients with COVID-19 remain asymptomatic or have mild symptoms throughout the disease course, but some patients present with severe symptoms, including acute respiratory distress syndrome [2–4]. In addition to underlying comorbidities, excessive inflammations associated with elevated procalcitonin, C-reactive protein (CRP), D-dimer, and lactate dehydrogenase are a poor prognostic factors for patients with COVID-19 [5,6]. Therefore, how to ameliorate excessive inflammation to improve the clinical outcomes of patients with COVID-19 has become a critical issue. However, only 2 anti-inflammatory agents, namely corticosteroid and anti-interleukin-6, have been confirmed to have clinical efficacy in reducing mortality among hospitalised patients with COVID-19 and have been recommended in clinical practice [7–11].

Although many medications, such as selinexor, allopurinol, ursolic acid can exhibit anti-inflammation activity and are repurposed for the treatment of SARS-CoV-2 infections [12–14], a readily available and inexpensive medication for complication prevention in patients with COVID-19 is still urgently required. Colchicine—an anti-inflammatory agent that has potent activity in the nucleotide binding domain–like pyrin domain 3 inflammasome, cellular adhesion molecules, and inflammatory chemokines—has been repurposed as a promising agent in this clinical setting [15–19]. Clinically, a case series of five patients showed that the use of colchicine and doxycycline combination could be associated with marked improvements in the clinical, laboratory and radiological outcomes in patients with COVID-19 pneumonia [20]. Several clinical studies have been conducted to investigate the clinical efficacy of colchicine in COVID-19 treatment and to demonstrate the benefits of colchicine [21–25]. A cross-sectional study involving 301 adults with COVID-19 pneumonia reported that the mortality rate in the colchicine-treatment group was lower than that in the control group (9.6% vs. 14.6%, *p* = .179) [22]. Another large clinical trial conducted by Tardif et al. demonstrated that colchicine led to a lower rate of composite variables, death or hospital admission, than did placebo among 4159 patients with confirmed COVID-19 in a community setting (odds ratio [OR], 0.75; 95% CI, 0.57–0.99; *p* = .042) [21]. By contrast, several clinical studies have shown that colchicine is not beneficial in COVID-19 treatment [26–28]. The RECOVERY trial revealed that colchicine was not associated with reductions in the 28-day mortality rate, duration of hospital stay, or risks of progression to invasive mechanical ventilation (MV) and death [28]. To solve this controversy, we conducted this systematic review and meta-analysis of randomised controlled trials (RCTs) to provide robust and up-to-date evidence of the clinical efficacy and safety of colchicine for patients with COVID-19.

## 2. Methods

### 2.1. Search strategy

We searched PubMed, Web of Science, Ovid MEDLINE, the Cochrane Library, Embase, and Clinicaltrials.gov for relevant articles from inception to November 12, 2021. The following search terms were used: “COVID-19,” “coronavirus infections,” “corona virus,” “corona infection,” “sars-cov-2,” and “colchicine”. Only RCTs that assessed the clinical efficacy of colchicine in the treatment of patients with COVID-19 were included. Furthermore, we manually searched for additional eligible articles in the reference lists of selected articles. To prevent bias, two authors (SHL and CCL) independently screened the literature and identified publications. A third author (SPC) was consulted in case of disagreement. This study was conducted in accordance with the Preferred Reporting Items for Systematic Reviews and Meta-Analyses guidelines [29]. The protocol of the systematic review and meta-analysis was registered at PROSPERO [CRD42021293450].

### 2.2. Eligibility criteria

Studies were included if they met the following criteria: (1) patients with COVID-19 included; (2) colchicine used for intervention; (3) a placebo or standard care used as the comparator; (4) RCT design; and (5) study outcomes included clinical efficacy and safety. The exclusion criteria included cohort studies, nonhuman studies, reviews, meta-analyses, studies without adequate data for outcome analysis, and poster or conference abstracts were excluded. In addition, if the colchicine group involved other agents that were not used in the control group, the corresponding studies were also excluded.

### 2.3. Data extraction

The following data were extracted separately by 2 authors (CCL and LCL) from each included study: publication year, study design, colchicine regimen, clinical outcomes, and adverse event (AE) risk. A third author (CKH) was consulted if the extracted data were inconsistent. The primary outcome was all-cause mortality. The secondary outcomes were the need for non-invasive ventilation (NIV) or MV, length of hospital stay, and the risks of AEs. Any AE was defined as an event that emerges during treatment, having been absent pre-treatment, or worsens relative to the pre-treatment state. Serious AE was defined as an AE that results in death, is life-threatening, requires inpatient hospitalisation or extends a current hospital stay, results in an ongoing or significant incapacity or interferes substantially with normal life functions.

### 2.4. Data analysis

Two investigators (SHL and SPC) independently assessed the risk of bias for each of the included studies by using the Cochrane risk-of-bias tool 2.0 [30]. Furthermore, we used Review Manager (version 5.3; Nordic Cochrane Centre, Copenhagen, Denmark) for statistical analyses. The degree of heterogeneity was evaluated using Q statistics generated from the *χ*^2^ test, and the *I*^2^ measure was used to assess statistical heterogeneity. Heterogeneity was considered significant when *p* < .10 or *I*^2^ > 50%. A fixed-effects model was applied for homogeneous data, and a random-effects model was applied for heterogeneous data. We calculated the pooled ORs and mean difference (MD) with 95% CIs for analysis of the outcomes of interest using the Mantel-Haenszel formula.

## 3. Results

### 3.1. Study selection

**Figure 1.**
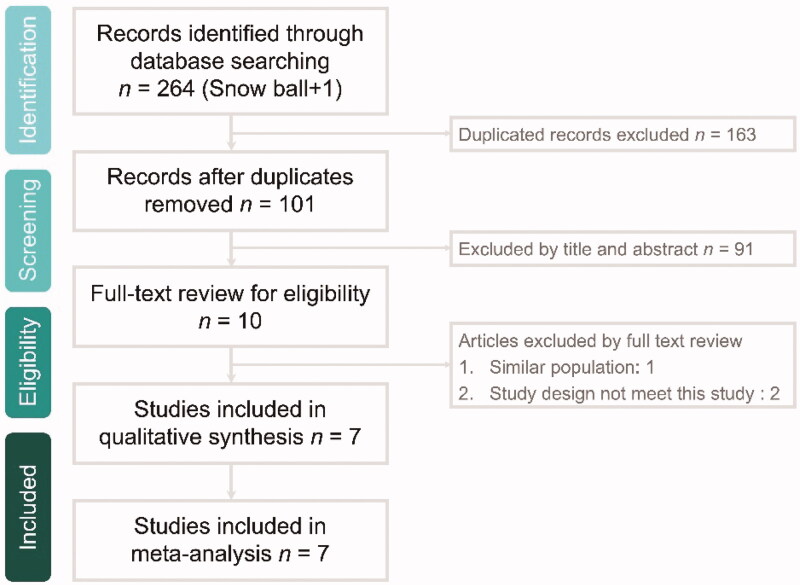
Flow diagram of study selection.

The online database search yielded 264 studies, of which 163 were duplicate studies and excluded. In addition, 91 studies were found to be either irrelevant after screening of titles and abstracts or with incomplete text. Furthermore, 3 studies were excluded (similar population: *n* = 1 and not RCTs: *n* = 2) after screening the full texts of 10 articles. Finally, 7 RCTs [21,23–28] were included in the meta-analysis (Figure 1 and Appendix 1).

### 3.2. Study characteristics

**Figure 2.**
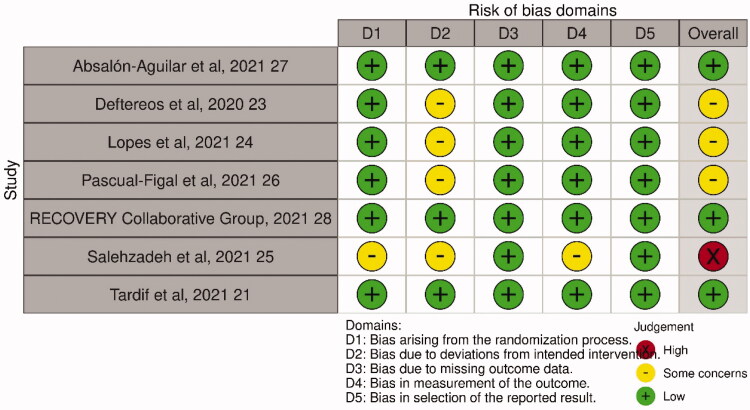
Summary of the risk of bias in each domain.

Among the 7 RCTs, 4 were multicenter studies [21,23,27,28] and 3 were single-centre studies [24–26] (Table 1). In addition, 2 were multinational studies [21,28]. and one each was conducted in Mexico [27]. Greece [23]. Brazil [24], Spain [26], and Iran [25], One study [21] focussed on nonhospitalized patients; the other 6 RCTs [23–28] enrolled hospitalised patients with COVID-19. Among the RCTs, the colchicine regimen varied, and the treatment duration ranged from 6 to 28 days. Overall, 16,024 patients were included in this study, with 7,794 in the study group receiving colchicine and 8,230 in the control group receiving placebo or standard treatment. Regarding the risk of bias, three studies [23,24,26] have bias due to deviations from intended interventions, and one study [25] has some concerns for multiple domains and its overall risk of bias was classified as high (Figure 2).

**Table 1.** Characteristics of included studies.

Study	Design	Sites	Patients	Regimen of colchicine	Comparator	Number of patients	
						Colchicine	Control
Absalón-Aguilar et al., [27]	Triple-blind parallel nonstratified placebo-controlled clinical trial	Multicenter in Mexico	Hospitalised patients with severe COVID-19: respiratory failure, respiratory rat*e* ≥30 bpm, oxygen saturatio*n* ≤93% at rest, PaO_2_/FiO_2_ ≤300 mmHg.	1.5 mg loading follow by 0.5 mg bid for 10 days	Placebo	56	60
Deftereos et al. [23]	open-label, randomised clinical trial	Multicenter in Greece	Hospitalised patients with COVID-19 and arterial oxygen partial pressure lower than 95 mmHg on room air	1.5 mg loading follow by 0.5 mg bid for as long as 3 weeks	Standard treatment	55	50
Lopes et al. [24]	randomised, double-blinded, placebo-controlled clinical trial	Single centre in Brazil	Hospitalised patients with moderate (pneumonia on image) to severe (respiratory rat*e* ≥30 bpm, oxygen saturatio*n* ≤ 93%) COVID-19	0.5 mg tid for 5 days, then 0.5 mg bid for 5 days	Standard treatment	36	36
Pascual-Figal et al. [26]	randomised, controlled and open-label clinical trial	Single centre in Spain	Hospitalised patients with COVID-19 and 7-points WHO clinical status of 3, 4 or 5.	1.5 mg loading dose, followed by 0.5 mg bid for one week and 0.5 mg qd for 28 days	Standard treatment	52	51
RECOVERY Collaborative Group [28]	randomised, controlled, open-label trial	Multicenter in multination	Hospitalised patients with COVID-19	1 mg loading followed by 500 μg bid for 10 days in total or until discharge	Standard treatment	5310	5730
Salehzadeh et al. [25]	randomised, double-blinded, clinical trial	Single centre in Iran	Hospitalised patients with COVID-19 and pulmonary involvement seen in CT scan	1 mg qd for 6 days	Placebo	50	50
Tardif et al. [21]	randomised, double-blind, adaptive, placebo-controlled trial	Multicenter in multination	Non-hospitalised patients with COVID-19 and at least one of the high-risks	0.5 mg bid for 3 days and then qd for 27 days	Placebo	2235	2253

### 3.3. Primary outcome

**Figure 3.**
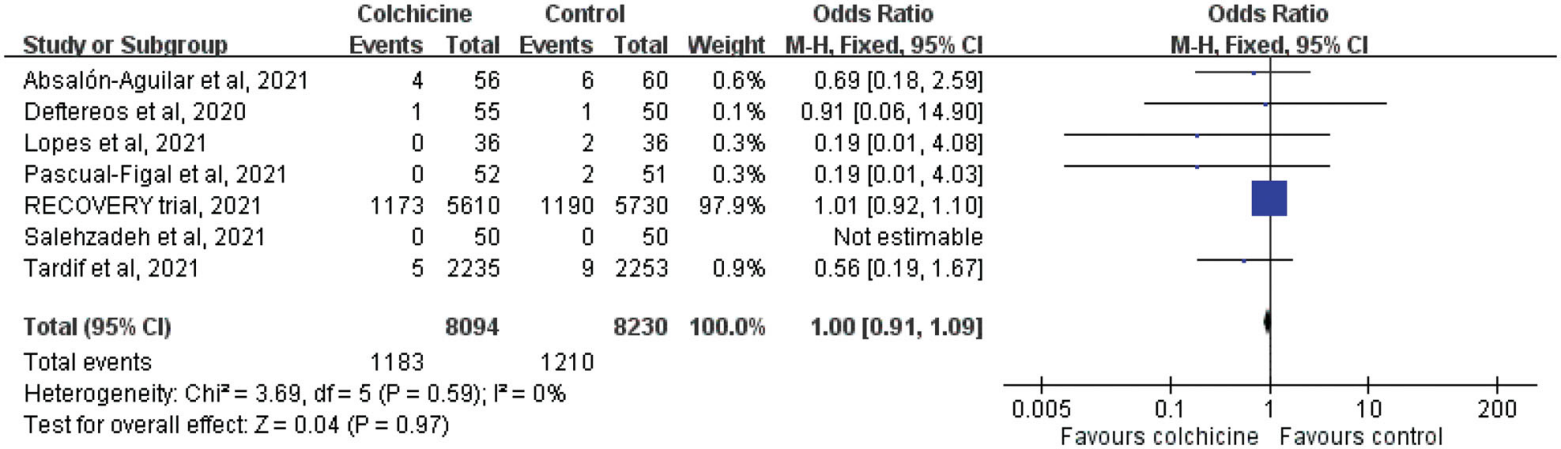
Forest plot of the mortality rate between colchicine and Control cohorts.

The mortality rate among patients who received colchicine was 14.6% (1183/8094), similar to that among the controls (14.7%, 1210/8230; OR, 1.00; 95% CI, 0.91–1.09; *I*^2^ = 0%; Figure 3). This difference remained insignificant in the leave-one-out sensitivity test, in which individual studies were randomly excluded. In the subgroup analysis of hospitalised patients, the study and control groups had a similar risk of death (OR, 1.00; 95% CI, 0.92–1.10; *I*^2^ = 0%).

### 3.4. Secondary outcomes

**Figure 4.**
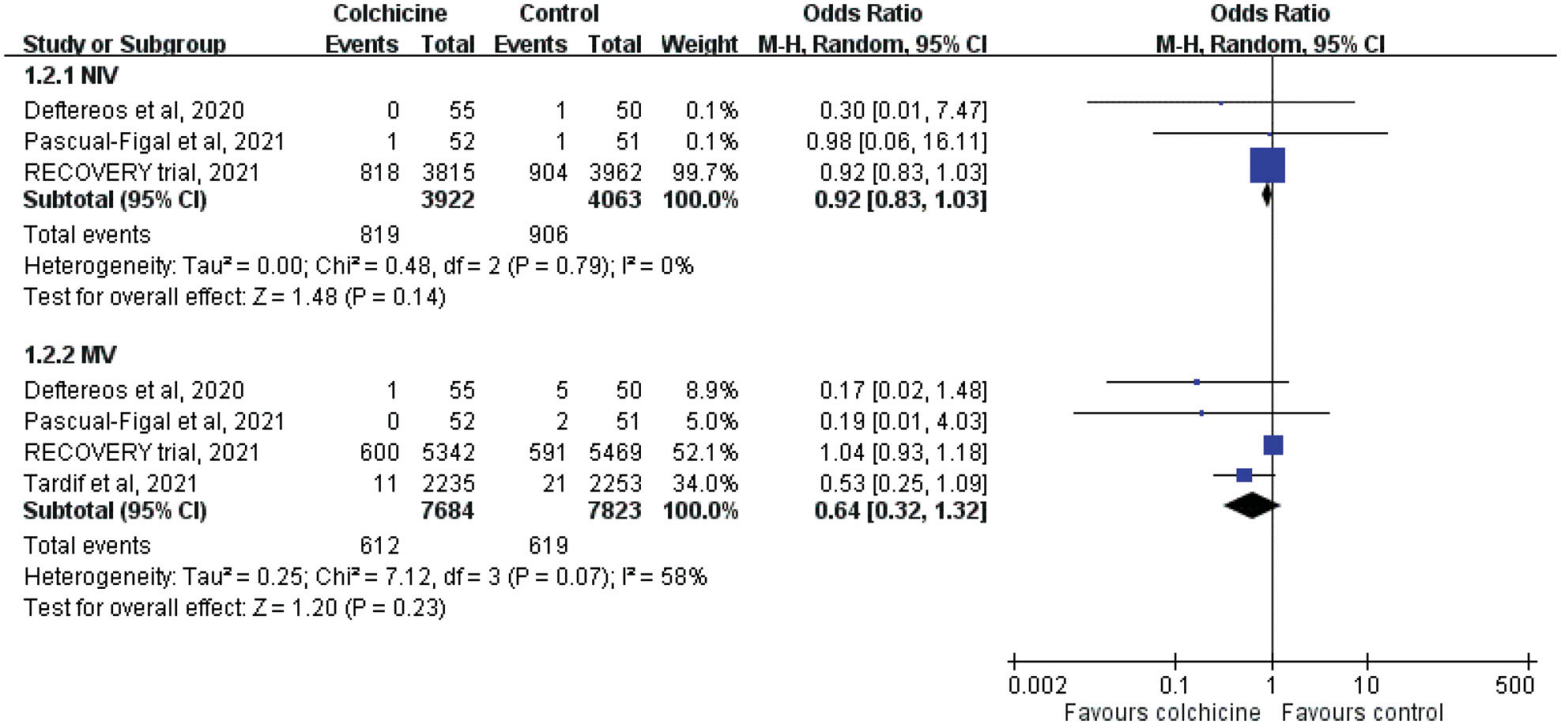
Forest plot of the need for non-invasive ventilation (NIV) and mechanical ventilation (MV) between colchicine and control cohorts.

**Figure 5.**
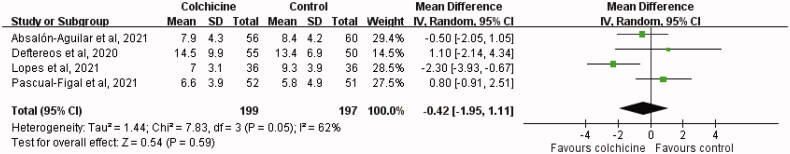
Forest plot of the length of hospital stay between colchicine and control cohorts.

Regarding the need for oxygen support, no significant difference was observed between the study and control groups in terms of the need for NIV (OR, 0.92; 95% CI, 0.83–1.03; *I*^2^ = 0%) or the need for MV (OR, 0.64; 95% CI, 0.32–1.32; *I*^2^ = 58%; Figure 4). In addition, no significant difference was observed in the length of hospital stay between the colchicine and control groups (MD, −0.42 days; 95% CI, −1.95 to 1.11; *I*^2^ = 62%; Figure 5).

**Figure 6.**
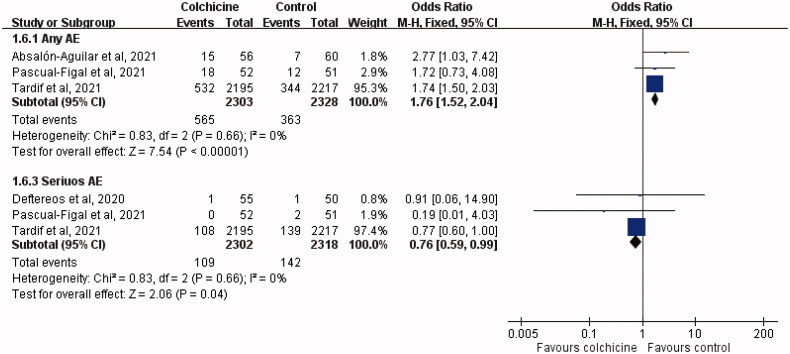
Forest plot of the comparison of the risk of adverse events (AEs) between colchicine and control cohorts.

**Figure 7.**
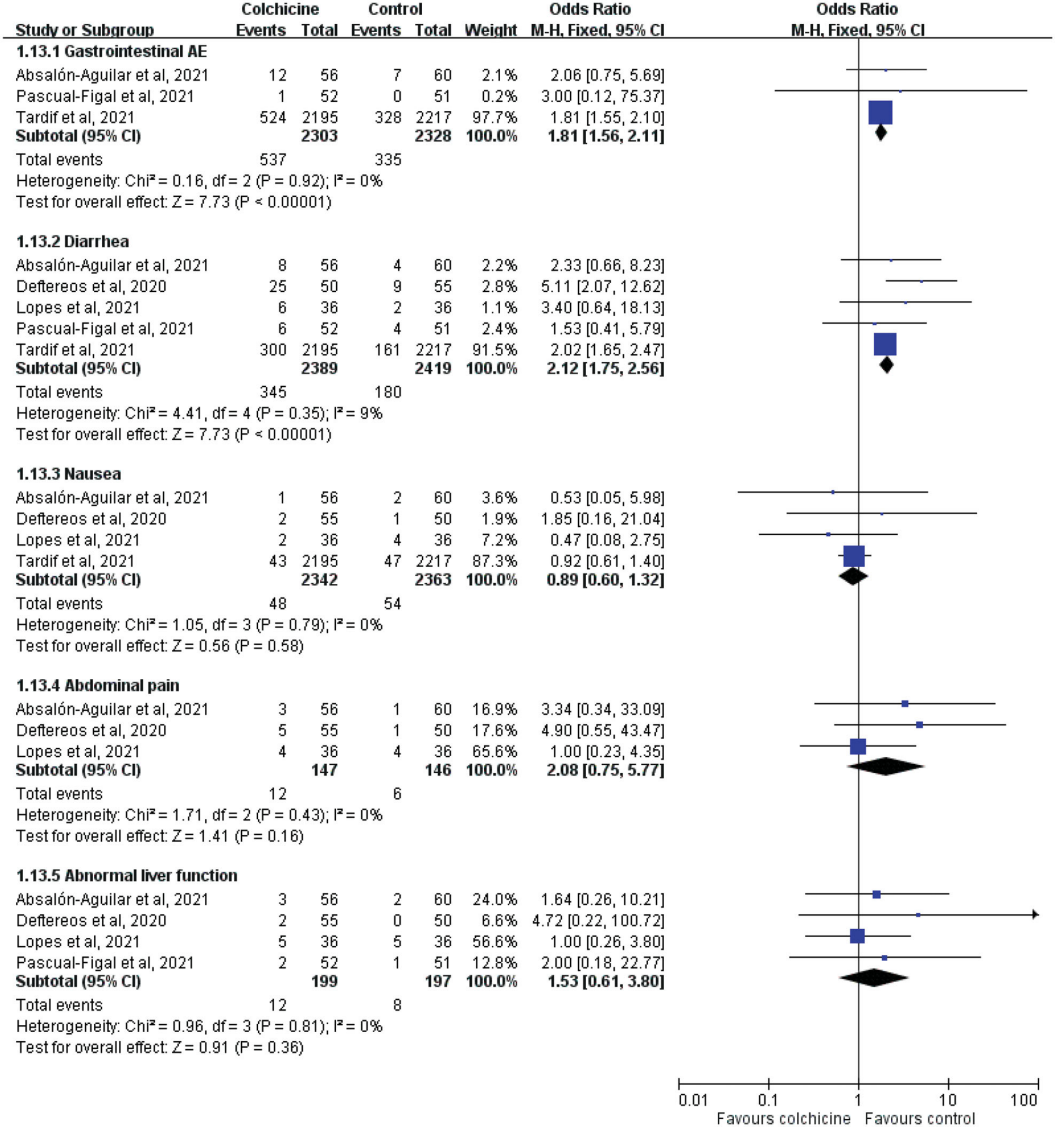
Forest plot of the comparison of the risk of specific adverse events (AEs) between colchicine and control cohorts.

The colchicine group was associated with higher risk of any AE than the control group (OR, 1.76; 95% CI, 1.52–2.04; *I*^2^ = 0%), but the risk of severe AE was lower in the colchicine group than in the control group (OR, 0.76; 95% CI, 0.59–0.99; *I*^2^ = 0%; Figure 6). Regarding specific AEs, colchicine was associated with significantly higher risks of gastrointestinal AE (OR, 1.81; 95% CI, 1.56–2.11; *I*^2^ = 0%) and diarrhoea (OR, 2.12; 95% CI, 1.75–2.56; *I*^2^ = 9%) (Figure 7). However, no significant difference was observed between the study and control groups in terms of the risks of nausea (OR, 0.89; 95% CI, 0.60–1.32; *I*^2^ = 0%), abdominal pain (OR, 2.08; 95% CI, 0.75–5.77; *I*^2^ = 0%), and abnormal liver function (OR, 1.53; 95% CI, 0.61–3.80; *I*^2^ = 0%) (Figure 7).

## 4. Discussion

In this meta-analysis, 7 RCTs [21,23–28] involving 16,024 patients were reviewed to investigate the efficacy and safety of colchicine in COVID-19 treatment. Overall, we found that colchicine does not confer an additional clinical benefit for patients with COVID-19, which was supported by the following evidence. First, we discovered that the mortality rate among patients receiving colchicine did not differ from that among those receiving placebo or standard care, and this result remained consistent in the leave-one-out sensitivity test and in the subgroup analysis of hospitalised patients with COVID-19. All these findings were based on an analysis of RCTs with low heterogeneity. Second, we found that the colchicine and control groups had similar risks in terms of the need for NIV or MV and length of hospital stay. These findings were consistent with recent studies [31–33], in which colchicine did not provide an additional effect on all-cause mortality, length of hospitalisation, ICU admission and MV. Although all this evidence does not support colchicine use for COVID-19 treatment, our findings should be interpreted with caution. The characteristic and severity of patients were heterogeneous, and the regimen of colchicine was not the same in each included study. Therefore, the pooled analysis did not find a significant difference between the colchicine and the control group.

However, our findings are different from some previous meta-analyses [34–37]. One meta-analysis including 9 studies and 5522 patients demonstrated that significantly lower mortality was observed in the colchicine group than in the control group (OR, 0.35; 95% CI, 0.25–0.48; *I*^2^ = 0%) [34]. A meta-analysis of 10 studies reported that colchicine therapy is associated with a decreased mortality rate in patients with COVID-19 (OR, 0.365; 95% CI, 0.555–0.748; *I*^2^ = 24%) [35]. The meta-analysis by Lien et al. demonstrated that patients with colchicine treatment had significantly decreased the risk of mortality (OR, 0.57, 95% CI, 0.38–0.87; *I*^2^ = 72%), but no significant difference was observed in the mortality rate in the subgroup analysis of 4 RCTs (OR, 0.80; 95% CI, 0.44–1.46; *I*^2^ = 33%) [37]. Most of the studies included in these meta-analyses were observational studies [34–37] and the difference between the findings of our study and those of previous meta-analyses [34–37] could be because we only included RCTs in our meta-analysis. In contrast, our findings were consistent with a recent meta-analysis of 10 RCTs by Kow et al. [38] and another meta-analysis of 6 RCTs by Mehta et al. [33]. However, some of the included studies in Kow et al’s meta-analysis [38] used colchicine combined with other treatments as an intervention. But in the present meta-analysis, the study used the colchicine group involved other agents as the intervention was excluded. Therefore, our findings could be representative of the pure effect of colchicine. In addition, we have a serious concern about the methodology of one study by Mareev et al. [39] included in the meta-analysis by Mehta et al. [33]. In this study by Mareev et al. [39], although 20 people were expected to be randomised in the control group, their enrolment was discontinued after the inclusion of 5 patients due to the risk of severe deterioration in the absence of anti-inflammatory treatment. Additional 17 patients, who had not received anti-inflammatory therapy when treated before the study, were included in the control group. Therefore, the present study did not include this study [39] as Mehta et al. [33].

In addition to clinical efficacy, we assessed the safety of colchicine in the treatment of patients with COVID-19. Compared with the control group, the colchicine group experienced more AEs, particularly gastrointestinal AEs. However, most of the AEs were mild to moderate in severity. Moreover, colchicine was associated lower risk of severe AE compared with the control. These findings indicate that colchicine is a tolerable agent for patients with COVID-19.

This study has several limitations. First, only 7 RCTs were included, but the samples in the RCTs were large. Second, 5 RCTs had small samples (<120) whereas 2 had much larger samples [21,28]; therefore, the results of these two trials may have carried much greater weight when obtaining outcomes in the present meta-analysis. However, we used the leave-one-out sensitivity test to assess the effect of individual studies, and the results remained consistent. Third, the colchicine regimen and the severity of the included patients varied between the studies, but most of the findings were based on analysis of data with low heterogeneity (*I*^2^ < 50%). Finally, most of the included RCTs have a high risk of bias which can also affect their results and our analyses in the present study.

## 5. Conclusion

Colchicine does not improve the following clinical outcomes of patients with COVID-19: mortality, need for NIV or MV, and length of hospital stay. Colchicine was found to be a safe agent for COVID-19 treatment. However, our findings based on a meta-analysis of RCTs do not support colchicine use in the treatment of patients with COVID-19.

## Data Availability

The authors confirm that the data supporting the findings of this study are available within the article.

## Appendix 1. Search strategy

**Table:**

PubMed search strategy – last searched on November 12, 2021		Results
1	Search: Colchicine[Title/Abstract]	16768
2	Search: ((((Covid-19[Title/Abstract]) OR (SARS-CoV-2[Title/Abstract])) OR (coronaviru[Title/Abstract])) OR (2019-nCoV[Title/Abstract])) OR (corona-virus[Title/Abstract])	798872
3	Search: random*[Title/Abstract]	1269649
4	((Colchicine[Title/Abstract]) AND (((((Covid-19[Title/Abstract]) OR (SARS-CoV-2[Title/Abstract])) OR (coronavirus[Title/Abstract])) OR (2019-nCoV[Title/Abstract])) OR (corona-virus[Title/Abstract]))) AND (Random*[Title/Abstract])	42

Web of Science search strategy – last searched on November 12, 2021		Results
1	Colchicine (Topic)	35853
2	Covid-19 (TOPIC) or SARS-CoV-2 (TOPIC) or coronavirus (TOPIC) or 2019-nCoV (TOPIC) or corona-virus (TOPIC)	278794
3	Random* (Topic)	3134873
4	#1 AND #2 AND #3	52

Ovid medline search strategy – last searched on November 12, 2021		Results
1	Colchicine.ab.	13984
2	(Covid-19 or SARS-CoV-2 or coronavirus or 2019-nCoV or corona-virus).ab.	144991
3	Random*.ab.	1230549
4	1 and 2 and 3	42

Cochrane Library search strategy – last searched on November 12, 2021		
1	(Colchicine):ti,ab,kw	1020
2	(Covid 19):ti,ab,kw OR (SARS CoV 2):ti,ab,kw OR (coronavirus):ti,ab,kw OR (2019 nCoV):ti,ab,kw OR (corona virus):ti,ab,kw	8425
3	(Random*):ti,ab,kw	1117428
4	#1 AND #2 AND #3	50

Embase – last searched on November 12, 2021		
1	colchicine:ti,ab,kw	21126
2	'covid 19′:ti,ab,kw OR 'sars cov 2′:ti,ab,kw OR coronavirus:ti,ab,kw OR '2019 ncov':ti,ab,kw OR 'corona virus':ti,ab,kw	203629
3	random*:ti,ab,kw	1727318
4	#1 AND #2 AND #3	42

Clinicaltrials.gov – last searched on November 12, 2021		
1	Colchicine | Covid 19	35
